# Modulation of Cardiac Gene Expression by anti-HMGB1 in a Model of Experimental Myocardial Infarction

**DOI:** 10.33549/physiolres.935718

**Published:** 2025-12-01

**Authors:** Martina CEBOVA, Andrej BARTA, Katarina BUJNOVA, Maria VOJTKOVA, Stanislava VRANKOVA, Jana KLIMENTOVA, Olga PECHANOVA

**Affiliations:** 1 Department of Neuro-Cardiovascular Interaction Institute of Normal and Pathological Physiology, Centre of Experimental Medicine Slovak Academy of Sciences, Bratislava, Slovak Republic; 2 Institute of Pathophysiology, Faculty of Medicine, Comenius University, Bratislava, Slovak Republic; 3 University of Economics in Bratislava, Faculty of Economic Informatics, Department of Statistics, Bratislava, Slovak Republic

**Keywords:** Myocardial infarction, High mobility group box 1, Nitric oxide, Inflammation

## Abstract

Myocardial infarction (MI) remains a major cause of morbidity and mortality. The ischemic myocardium undergoes necrosis and apoptosis, triggering inflammation and oxidative stress that drive fibrosis and heart failure. High-mobility group box 1 (HMGB1) protein acts as a key damage-associated molecular pattern, activating TLR4 and NFκB signaling to promote cytokine release and exacerbate injury. The present study investigated the role of HMGB1 in MI and its impact on inflammatory and redox-related pathways, focusing on the effects of HMGB1 blockade. Male WKY rats were divided into the following groups: sham, MI, and MI with anti-HMGB1 treatment (MI+aHMGB1). MI was induced in rats by coronary ligation followed by reperfusion, and the animals were evaluated seven days later. Plasma cytokines, total NOS activity and gene expression in the left ventricle were analyzed. MI significantly increased plasma TNFα and IL-6, while anti-HMGB1 treatment reduced both cytokines. *Hmgb1 mRNA* was markedly upregulated after MI and normalized by aHMGB1. MI suppressed *Nos3* gene expression and total NOS activity, both of which were restored by aHMGB1. *Tlr4* and *NFκB mRNA* levels were elevated after MI and remained high after HMGB1 inhibition, whereas *Nos2* and *IL-1β* gene expression declined. Antioxidant responses showed differential regulation: *Sod1* and *Sod2* were further upregulated by aHMGB1, *Gpx4* expression normalized, and lipid peroxidation was found to be partially attenuated. These findings indicate that HMGB1 is a key driver of post-infarction inflammation and oxidative injury. Its inhibition modulates cytokine production, restores redox balance, and enhances endothelial protection, suggesting a promising therapeutic target for limiting myocardial damage.

## Introduction

Myocardial infarction (MI) arises from the sudden interruption of coronary blood flow, which is most commonly caused by thrombosis over an atherosclerotic plaque. This leads to ischemia and subsequent necrosis of myocardial tissue. This acute event triggers a series of inflammatory, oxidative and reparative processes that determine the severity of cardiac injury and functional outcome. Despite the advances in reperfusion therapies and pharmacological treatments, MI remains a leading cause of global cardiovascular mortality, representing approximately 32 % of all deaths worldwide. Of these deaths, 85 % were due to heart attack and stroke [1]. Thus, a better understanding of the molecular mediators involved in these processes is critical for improving prognosis and developing new therapeutic strategies.

Among these mediators, high mobility group box 1 (HMGB1) has emerged as a key damage-associated molecular pattern (DAMP) protein with dual, time-dependent roles in myocardial injury and repair. Under physiological conditions, HMGB1 is a nuclear protein that stabilizes chromatin structure and regulates DNA replication, transcription, and repair [2]. However, following myocardial injury, HMGB1 is passively released from necrotic cells or actively secreted by immune cells, where it serves as an alarmin that binds to pattern recognition receptors such as Toll-like receptors 4 and 9 (TLR4, TLR9) [3]. These receptors initiate downstream signaling cascades including activation of nuclear factor kappa B (NFκB), a key transcriptional regulator of pro-inflammatory genes such as tumor necrosis factor α (TNFα) and interleukin-1β (IL-1β). These cytokines further amplify inflammation and promote cardiomyocyte apoptosis, fibrosis, and adverse extracellular matrix remodeling [4–6].

In parallel, myocardial ischemia and reperfusion injury disrupt the balance of nitric oxide synthase (NOS) isoforms. Inducible NOS (iNOS) is typically upregulated during the inflammatory response, generating excessive nitric oxide (NO) which reacts with reactive oxygen species (ROS) to form the toxic peroxynitrite [7]. Recent experimental studies have confirmed a significant increase in iNOS expression following myocardial infarction, primarily in the infarcted myocardium and peri-infarct regions [8]. This excessive iNOS induction has been linked to increased nitrosative stress, mitochondrial dysfunction and cardiomyocyte apoptosis [9]. Antioxidant or anti-inflammatory treatments have been shown to reduce myocardial injury and improve cardiac recovery [10]. Taken together, these findings emphasise the harmful impact of uncontrolled iNOS activation on post-infarction tissue damage and adverse cardiac remodeling. Conversely, endothelial NOS (eNOS) activity is often downregulated, which contributes to endothelial dysfunction and impaired vasodilation. This redox imbalance exacerbates myocardial injury, promoting microvascular obstruction and progressive tissue damage. Recent studies have shown that ischemia and reperfusion are associated with reduced eNOS expression and uncoupling, resulting in diminished nitric oxide bioavailability and increased oxidative stress [11–12]. Furthermore, experimental models have shown that chronic nitrate supplementation or NO-donor therapy can restore eNOS levels and improve post-infarction outcomes [8].

Oxidative stress plays a key role in post-infarction remodeling. The antioxidant enzymes superoxide dismutase (SOD) and glutathione peroxidase 4 (GPX4) play a critical role in neutralizing ROS and preventing lipid peroxidation. In experimental models, suppression of the GSH/GPX4 axis has been shown to promote ferroptosis during ischemia-reperfusion injury Recent studies have confirmed that GPX4 depletion leads to excessive lipid peroxidation, mitochondrial dysfunction and loss of cardiomyocyte viability [13]. Furthermore, activation of ferroptotic signaling has been identified as a key mechanism linking oxidative stress with maladaptive post-infarction remodeling and heart failure progression [14]. Pharmacological enhancement of SOD and GPX4 activity or glutathione precursor supplementation has been shown to mitigate ferroptosis and improve cardiac functional recovery following myocardial infarction [15–16]. Taken together, these findings highlight the therapeutic potential of targeting redox homeostasis to limit oxidative injury and preserve myocardial integrity.

Taken together, the post-MI environment is shaped by a complex interplay of HMGB1 signaling, TLR-mediated immune activation, NOS dysregulation, and oxidative stress. Dysregulation of antioxidant defenses, particularly GPX4, further perpetuates cellular damage, creating a self-sustaining cycle that contributes to adverse cardiac remodeling and heart failure progression.

In this study, we focused on dissecting the molecular mechanisms by which HMGB1 influences gene expression during the early post-infarction phase. Specifically, we examined the expression of genes involved in inflammation (NFκB, TNFα, IL-1β), oxidative stress (SOD, GPX4), and vascular homeostasis (eNOS, iNOS), as well as innate immune activation through TLR4. By evaluating these molecular markers, we aimed to clarify the role of HMGB1 in coordinating inflammatory and oxidative responses, and to explore its potential as a therapeutic target in the context of myocardial infarction.

## Materials and Methods

### Animals and treatment

12-week-old male Wistar Kyoto rats were used in the study. The animals were kept in controlled environmental conditions, with temperature of 22 ± 2 °C, a relative humidity at 55 ± 10 %, and a standard 12-hour light/dark cycle. The rats were obtained from Velaz Laboratories (Czech Republic). All experimental procedures complied with institutional regulations and were approved by the State Veterinary and Food Administration of the Slovak Republic (protocol numbers Ro-4430/13-221), as well as by the Ethics Committee of the Centre of Experimental Medicine, Slovak Academy of Sciences. The experiments were conducted in accordance with the European Directive 2010/63/EU on the protection of animals used for scientific purposes and the European Convention for the Protection of Vertebrate Animals. Reporting of the study followed the ARRIVE guidelines (https://arriveguidelines.org)

The animals were randomly assigned to three groups: (1) sham-operated 12-week-old male WKY rats (sham); (2) age-matched male WKY rats subjected to experimental myocardial infarction (MI), and (3) age-matched male WKY rats with myocardial infarction treated with anti-HMGB1 (Abcam, Cambridge, UK) at a dose of 10 μl (MI + aHMGB1). Each group consisted of 6 animals. All rats had unrestricted access to drinking water and standard laboratory chow (Altromin 1324P). Blood pressure was recorded in pre-warmed animals by noninvasive tail-cuff plethysmography using the MRBP system (IITC Life Science), both prior to surgery and again 7 days following myocardial infarction.

### Experimentally induced myocardial infarction

Experimental MI was induced in 12-week-old normotensive WKY rats by ligation of the left descending coronary artery, following the protocol described by Kosutova et al. [17]. Briefly, preoperative analgesia was achieved by administering butorphanol (2 mg/kg, s.c.), meloxicam (2 mg/kg, s.c.), and 5 ml saline with 5 % glucose (s.c.). Anaesthesia was then induced with tiletamine–zolazepam (30 mg/kg, i.p.), after which the rats were intubated and ventilated at a rate of 70 breaths per minute using a pressure-controlled rodent respirator. Throughout surgery, body temperature was maintained at 37 °C using a heating pad. A left lateral thoracotomy was performed via the fifth intercostal space. After opening the pericardium, the left coronary artery was reversibly ligated with 5-0 silk suture (Ethicon, San Lorenzo, USA) approximately 2 mm from its origin. Successful MI induction was confirmed by electrocardiography (ECG). Twenty minutes after ligation, but before reperfusion, 10 μl of anti-HMGB1 protein solution was injected directly into the myocardium. The thoracotomy was then closed in layers using Vicryl 4-0 SH-1 and Prolene 8-0 CC (Ethicon, San Lorenzo, USA). Sham-operated rats underwent the same surgical protocol except for coronary ligation. ECG recordings were obtained from four subcutaneous needle electrodes positioned at standard axillary and groin sites with the animal in a supine position (Rodent Surgical Monitor+, Indus Instruments, Webster, Texas, USA). Seven days post-MI, the animals were euthanized with an overdose of anesthetic, and their body weight (BW), heart weight (HW), and kidney weight (KW) were recorded. Plasma levels of TNFα and IL-6 were measured using the Bio-Plex Pro™ Rat Cytokine Assay Kit (Bio-Rad, Hercules, CA, USA), while cardiac troponin I was quantified with a Rat Cardiac Troponin I SimpleStep ELISA Kit (Abcam, Cambridge, UK).

### Total NOS activity

Total NOS activity was determined in 20 % crude tissue homogenates from the whole hearts by measuring the formation of [^3^H] L-citrulline from [^3^H] L-arginine (ARC, St. Louis, MT, USA) as previously described by Cebova et al. [18]. The [^3^H] L-citrulline content was measured by liquid scintillation counting using a Quanta Smart TriCarb Liquid Scintillation Analyser (PerkinElmer, Waltham, MA, USA). NOS activity is expressed as picokatal per gram of protein (pkat/g protein).

### RNA isolation and determination of gene expression

The gene expression levels of high mobility group box 1 (HMGB1), endothelial NOS (eNOS), inducible NOS (iNOS), superoxide dismutase (SOD), toll-like receptor 4 (TLR4), nuclear factor-κB p65 (RelA), interleukin 1 β (IL-1β), tumor necrosis factor α (TNFα), glutathione peroxidase 4 (GPX4) and the 60S ribosomal protein RPL10a (Rpl10a) in the left ventricle were determined by using two-step reverse transcription quantitative polymerase chain reaction (RT–qPCR). The total RNA of the left ventricle was isolated using TRIzol™ RNA Isolation Reagent (Invitrogen™, Thermo Fisher Scientific, Waltham, MA, USA) according to the manufacturer’s protocols. The amount and purity of total isolated RNA were spectrophotometrically quantified at 260/280 and 260/230 nm using a NanoDrop spectrophotometer (Thermo Scientific, Waltham, MA, USA). Reverse transcription was performed using 1 μg of total RNA from each sample and an Eppendorf Mastercycler (Hamburg, Germany) and iScript-Reverse Transcription Supermix (Bio-Rad, Hercules, CA, USA) according to the manufacturer’s protocols. Gene-specific primers were designed using the PubMed program (Primer-BLAST) and a database (Gene). The DNA sequences and melting temperatures of the primers used, the sizes of the amplicons and the reference numbers of the templates are given in Table 1. The PCRs were conducted in a final volume of 20 μL containing 2 μL of 5-fold diluted template cDNA, 10 μL of SsoAdvanced mix (SsoAdvanced Universal SYBR Green Supermix, Bio-Rad, Hercules, CA, USA), 1.5 μL of each the forwards and reverse primer (Metabion, Germany, 4 μmol/L), and 5 μL of RNase-free water (Sigma–Aldrich, Germany). The thermal cycling conditions were as follows: (1) 95 °C for 30 s and (2) 40 cycles of (a) 95 °C for 10 s and (b) the optimal annealing temperature (on the basis of the selected primer, see Table 1) for 20 s. Finally, melting curves for amplicon analyses were constructed in the range of 60–95 °C at a rate of 1 °C/5 s. RT–qPCR was performed using a CFX96 Real-Time PCR detection system (Bio-Rad, Hercules, CA, USA) and evaluated by Bio-Rad CFX Manager software 2.0 (Bio-Rad, Hercules, CA, USA). The expression of each gene was determined in 8 rats. The quantities (Ct values) of the target genes were normalized to the quantity (Ct) of the housekeeping gene (Rpl10a). Relative mRNA expression was calculated using the 2^−ΔΔCt^ method.

### Measurement of the conjugated diene content

The extent of lipid peroxidation was determined by measuring the levels of conjugated dienes in lipid extracts derived from the left ventricle. The tissue was initially homogenised in a solution of 4 % sodium chloride containing 15 mmol/L EDTA. Subsequently, lipids were extracted using a chloroform–methanol mixture in a 1:1 volume ratio. After phase separation, the chloroform layer was evaporated using a stream of nitrogen gas, and the resulting lipid residue was reconstituted in cyclohexane (PanReac AppliChem, Darmstadt, Germany). Spectrophotometric analysis of conjugated dienes was performed at a wavelength of 233 nm using a NanoDrop One device (Thermo Fisher Scientific, Waltham, MA, USA). The quantification was based on an extinction coefficient of ɛ = 29,000 L·mol^−1^·cm^−1^, and the results were normalized to tissue mass and reported in nanomoles per gram.

### Statistical analysis

The data are expressed as the mean ± S.E.M. values. The statistical analysis was conducted using a one-way analysis of variance (ANOVA) and the Bonferroni post hoc correction. Correlations between variables were evaluated by Peterson correlation coefficient (r). Statistical significance was considered to have been achieved at P<0.05. The data were analysed using GraphPad Prism v.10.1.2 (GraphPad Software, Inc., San Diego, CA, USA) and SAS Enterprise Guide, version 7.12. SAS Institute Inc., Cary, NC, USA.

## Results

### Hemodynamic parameters and biomarkers

Following coronary artery ligation, no significant differences were observed in basic morphometric parameters (including body weight, heart weight, or kidney weight) when comparing sham-operated controls with MI animals, and MI animals treated with anti-HMGB1 protein. Similarly, no group-dependent differences were detected in tibia length, a parameter commonly used for normalization in experimental cardiology. The calculated heart-to-body weight ratio also remained consistent across all experimental groups. Notably, administration of anti-HMGB1 protein had no measurable impact on any of these parameters, suggesting that myocardial infarction and HMGB1 inhibition do not affect systemic growth indices or organ weight distribution within the 7-day post-infarction period.

**Fig. 1:**
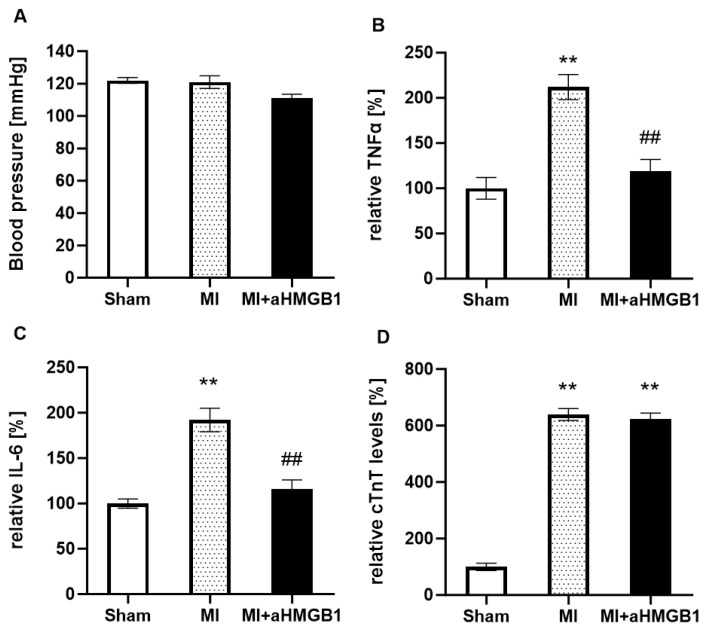
Blood pressure (BP) (**A**), relative tumor necrosis factor alpha (TNFα) (**B**), interleukin 6 (IL-6) (**C**) and cardiac troponin T (cTnT) (**D**) of sham-operated WKY rats (Sham), WKY rats with experimentally induced myocardial infarction (MI), and WKY rats with experimentally induced myocardial infarction and anti-HMGB1 administration (MI + aHMGB1). Values represent relative TNFα, IL-6 and cTnT levels. Data are expressed as percent changes relative to the Sham group (set to 100 %). ** p < 0.01 vs. Sham; ## p < 0.01 vs. MI; n = 6.

In the sham-operated WKY rats, baseline blood pressure, cytokine concentrations, and cardiac troponin levels remained within the expected physiological range. Myocardial infarction induced by coronary ligation did not result in any significant changes of blood pressure in either of two experimental groups (with or without aHMGB1) compared to the sham controls (Fig. 1A). However, a significant increase in the circulating levels of the pro-inflammatory cytokines was observed, with TNFα and IL-6 levels rising approximately twofold compared with the sham-operated group, indicating an enhanced systemic inflammatory response following myocardial injury. Administration of anti-HMGB1 protein effectively attenuated this inflammatory response, as reflected by a significant reduction of both TNFα and IL-6 towards values comparable with the sham-operated group (Fig. 1B,C). Cardiac troponin level, a marker of myocardial injury, was increased almost sixfold after infarction compared with sham animals. Treatment with anti-HMGB1 did not modify troponin concentrations (Fig. 1D).

### Total NOS activity and gene expression of Nos3, Nos2 and Hmgb1

**Fig. 2:**
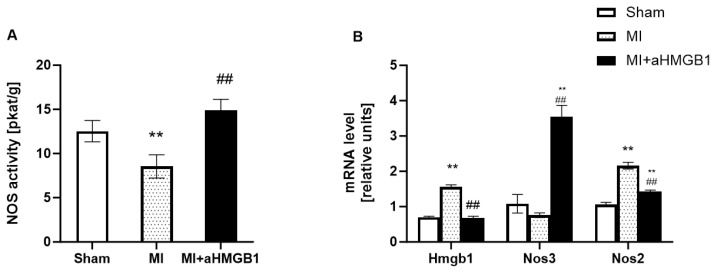
Total nitric oxide synthase activity (NOS) (**A**), gene expression of *Hmgb1*, *Nos3* and *Nos2* (**B**) in the heart of sham-operated WKY rats (Sham), WKY rats with experimentally induced myocardial infarction (MI), and WKY rats with experimentally induced myocardial infarction administered anti-HMGB1 (MI + aHMGB1). ** p < 0.01 vs. Sham; ## p < 0.01 vs. MI; n = 6. The data are the means ± SEMs.

Total NOS activity was significantly lower in the experimental myocardial infarction group than in the sham-operated control group (p < 0.05) (Fig. 2A). Myocardial infarction also tended to decrease expression of *Nos3* (p < 0.07), on the other hand significantly increase the expression of *Nos2* genes (p < 0.01) compared to the sham operated control (Fig. 2B). Interestingly, the administration of the anti-HMGB1 protein (MI+aHMGB1) led to a significant increase in the total NOS activity compared with sham controls as well as with animals after MI (p < 0.01), suggesting modulation via HMGB1 blockade (Fig. 2A). Anti-HMGB1 treatment resulted in a threefold increase in *Nos3* mRNA levels compared to the control group. In contrast, *Nos2 mRNA* expression was reduced after aHMGB1 administration, but it remained higher than that in the control group (Fig. 2B). Analysis of gene expression showed a significant upregulation of *Hmgb1* mRNA levels in the myocardium of animals subjected to myocardial infarction compared to sham-operated controls. This confirms the activation of HMGB1 signaling following ischaemic injury. Notably, treatment with the anti-HMGB1 antibody effectively suppressed this response, reducing *Hmgb1* gene expression to levels similar to those observed in the control group (Fig. 2B).

### Expression of genes involved in antioxidant defence and inflammatory responses

**Fig. 3:**
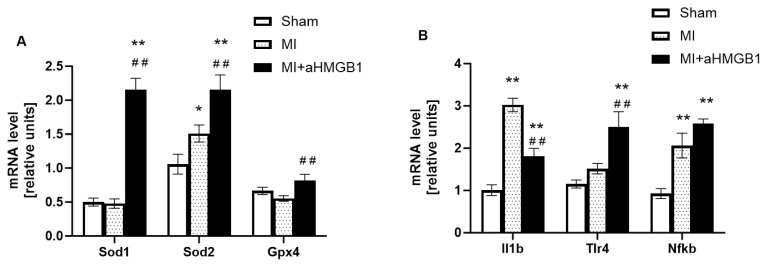
Gene expression of *Sod1*, *Sod2* and *Gpx4* (**A**) and *IL-1β*, *Tlr4* and *Nfκb* (**B**) in the heart of sham-operated WKY rats (Sham), WKY rats with experimentally induced myocardial infarction (MI), and WKY rats with experimentally induced myocardial infarction administered anti-HMGB1 (MI + aHMGB1). * p < 0.05, ** p < 0.01 vs. Sham; ## p < 0.01 vs. MI; n = 6. The data are the means ± SEM.

The expression of *Sod1*, which encodes cytosolic superoxide dismutase, did not change following myocardial infarction compared to the control group. However, treatment with anti-HMGB1 resulted in significant upregulation of *Sod1* gene expression in comparison to both MI groups (p < 0.01; Fig. 3A). This suggests that HMGB1 inhibition enhances the body’s antioxidant defenses and promotes the detoxification of superoxide radicals. On the other hand, MI animals exhibited increased *Sod2* gene expression, the mitochondrial isoform of superoxide dismutase, compared with sham controls (p < 0.05; Fig. 3A), indicating the activation of mitochondrial antioxidant responses in the post-ischaemic myocardium. The anti-HMGB1 administration further increased this, implying a potential synergistic effect on the regulation of mitochondrial oxidative stress (p < 0.01). In contrast, *Gpx4* gene expression, slightly decreased following myocardial infarction (p < 0.08), reflecting impaired antioxidant capacity. Anti-HMGB1 treatment restored *Gpx4* gene expression towards baseline levels, suggesting that HMGB1 inhibition mitigates oxidative lipid damage and contributes to redox homeostasis after MI (p < 0.01; Fig. 3A).

Significant upregulation of *IL-1β*, *Tlr4* and *NFκB* gene expression was observed in the myocardial infarction group (p < 0.01 or p < 0.05) compared with the sham-operated controls, indicating a strong inflammatory response following MI. The administration of anti-HMGB1 had differential effects on these genes. Although *IL-1β* expression decreased significantly compared to the MI group, suggesting partial attenuation of cytokine-mediated inflammation, both *Tlr4* and *NFκB* expression levels increased further after anti-HMGB1 treatment (Fig. 3B).

**Fig. 4:**
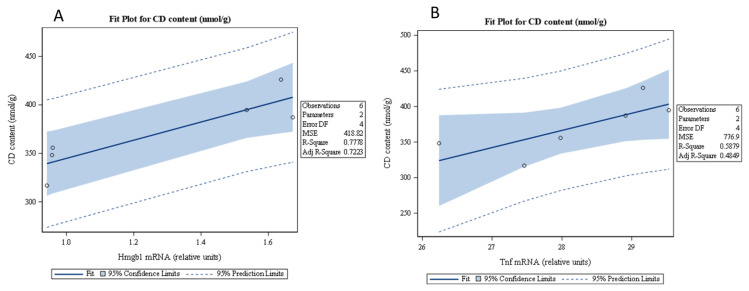
The correlations of Hmgb1 gene expression with CD content (**A**), Tnfα gene expression correlations with CD content (**B**) of sham-operated WKY rats (Sham), WKY rats with experimentally induced myocardial infarction (MI), and WKY rats with experimentally induced myocardial infarction administered anti-HMGB1 (MI + aHMGB1). ** p < 0.01 vs. Sham; ## p < 0.01 vs. MI; n = 6.

A significant increase in the concentration of conjugated diene was observed in the MI group compared to the sham controls (p < 0.01), suggesting increased lipid peroxidation and oxidative stress following myocardial infarction. Treatment with anti-HMGB1 reduced this elevation to a certain extent, demonstrating a clear attenuation of oxidative damage. While conjugated diene levels remained higher than in the control group, the decrease relative to untreated MI animals suggests that HMGB1 contributes to post-infarction oxidative injury, and that inhibiting it exerts a protective effect against lipid peroxidation. (Fig. 4A). Further statistical analysis revealed a positive correlation between the expression *Hmgb1* and *Tnfα* genes and the content of CD associated with oxidative damage (Fig. 4B,C).

## Discussion

Inflammation and myocardial fibrosis are recognized as key pathological drivers of heart failure (HF) progression following myocardial infarction [19]. Persistent inflammatory activity is associated with worse clinical outcomes and the exacerbation of HF severity. Following myocardial injury, a range of pro-inflammatory mediators, including IL-6, IL-1β, TNFα and MCP-1, are released and contribute to the activation of immune cells and amplification of the inflammatory cascade [20]. Although inflammation is crucial for initiating tissue repair, prolonged or excessive activation during the recovery period following a heart attack can disrupt cardiac homeostasis, promote adverse ventricular remodeling and contribute to the progression of heart failure [21]. Therefore, strategies aimed at modulating or resolving post-infarction inflammation represent a promising therapeutic approach for improving cardiovascular outcomes. In our study, we investigated the molecular alterations and intracellular signaling cascades that occur in rats that have undergone myocardial infarction. We paid special attention to the ways in which these pathways may be modulated in an experimental setting. Our results demonstrate that neither myocardial infarction nor anti-HMGB1 treatment influenced basic morphometric parameters, including body weight, heart weight, kidney weight, tibia length, or the heart-to-body weight ratio within the 7-day post-infarction period. These findings suggest that systemic growth indices and gross organ weights remained stable across groups, and that the observed biochemical changes were not affected by variations in overall somatic growth or organ mass.

No significant differences in blood pressure were observed between sham operated animals and MI rats. This finding is in contrast to the results of earlier studies [22, 23], which reported significant post-infarction hypotension, presumably due to the use of permanent coronary ligation models that result in larger infarcts and more severe myocardial dysfunction. Conversely, transient ligation with reperfusion, as employed in the present study, has been shown to preserve cardiac performance and limit systemic pressure decline. Furthermore, the administration of anti-HMGB1 did not significantly alter BP values, suggesting that the HMGB1 protein does not play a major regulatory role in systemic blood pressure under these experimental conditions. The temporal aspect of blood pressure measurement is another crucial factor. It is important to note that early postoperative recordings are often influenced by anesthesia and perioperative instability, which can transiently lower blood pressure. Subsequent measurements, however, have been shown to better reflect cardiovascular adaptation and may reveal persistent hypotension in animals with extensive myocardial injury [22]. As the measurements were taken seven days after surgery, at a point beyond the acute phase, the effects of the anesthetic were minimal. Collectively, these methodological discrepancies likely underpin the absence of blood pressure fluctuations in our study, underscoring the assertion that post-infarction hemodynamic responses are profoundly model- and time-dependent.

Systemic inflammatory response propagating beyond the local injury to encompass the entire organism. Ischemic necrosis of cardiomyocytes results in the release of DAMPs, including HMGB1, which in turn promotes the production of pro-inflammatory cytokines, including TNF-α and IL-6. These cytokines have been demonstrated to stimulate not only local immune responses but also to exert widespread systemic effects, including the enhancement of bone marrow activity, the initiation of the acute-phase response in the liver, and the induction of endothelial activation. While inflammation is an integral component of tissue repair, persistent or uncontrolled inflammation following myocardial infarction can result in maladaptive cardiac remodeling, diminished cardiac function, and the eventual onset of heart failure [24, 25]. Elevated levels of cytokines such as TNF-α and IL-6 are sustained for several days post-MI, reflecting ongoing immune activation. It is noteworthy that the inhibition of HMGB1 results in a substantial reduction of this cytokine response, thereby substantiating the hypothesis that HMGB1 plays a pivotal role in instigating the inflammatory cascade following myocardial infarction. The elevation of troponin after myocardial infarction, a key indicator of cardiac myocyte injury, was unaffected by anti-HMGB1 treatment. This finding suggests that HMGB1 inhibition may have minimal effect on early cell death following infarction, or that the specific dosage and timing of the treatment were insufficient to prevent troponin release.

Following myocardial infarction, there is a marked elevation in the gene expression of *Hmgb1*, a key player in the inflammatory response and tissue damage. This upregulation of HMGB1 has been demonstrated in various studies, thus highlighting its role as a damage-associated molecular pattern that promotes the inflammatory cascade and myocardial injury post-MI [26]. Following the administration of anti-HMGB1, levels of *Hmgb1* return to the baseline level. This finding supports the hypothesis that the inhibition of HMGB1 has a potential therapeutic role in reducing inflammation and myocardial damage following ischemic injury. The present study also encompassed an evaluation of the mRNA expression levels of genes implicated in nitric oxide signaling. Gene expression analysis revealed significant upregulation of *Nos3* and downregulation of *Nos2* in the aHMGB1-treated group compared to the MI-only group. Notably, *Nos3* expression was found to be three times higher in the aHMGB1 group, directly correlating with the total nitric oxide synthase activity. This finding suggests that anti-HMGB1 treatment exerts a dual effect on systemic inflammation, modulating it in addition to enhancing nitric oxide signaling. Nitric oxide signaling has been demonstrated to play a pivotal role in endothelial function, vasodilation, and myocardial perfusion post-MI. The present findings are consistent with those of earlier studies, which have demonstrated that increased endothelial nitric oxide synthase (eNOS) activity is associated with enhanced myocardial recovery and reduced ischemic damage [27].

The administration of aHMGB1 has been demonstrated to result in a significant increase in the expression of antioxidant enzymes, including *Sod1*, *Sod2*, and *Gpx4*. In this instance, the inhibition of HMGB1 may exert an indirect effect on antioxidant mechanisms through a reduction in ROS accumulation and inflammatory responses, the restoration of antioxidant enzyme activity (SOD and GPX4), and the promotion of mitochondrial biogenesis and function. The potential for these effects to be mediated by the activation of transcription factors such as Nrf2 is a hypothesis that has been postulated [28–30]. These transcription factors have been shown to regulate the expression of numerous antioxidant genes. Increased antioxidant activity has been reported to be related to the level of conjugated dienes, which are markers of lipid peroxidation and oxidative stress. These have been shown to be significantly reduced in the heart following anti-HMGB1 treatment. This finding also indicates a systemic antioxidant effect, in addition to that mediated by the reduction in HMGB1-driven inflammation. It is noteworthy that there was no significant variation in CD levels between the MI and sham groups.

The marked upregulation of *IL-1β*, *Tlr4*, and *NFκB* gene expression after myocardial infarction is consistent with the established role of the HMGB1/TLR4/NFκB axis. In conditions of ischemic stress, the release of HMGB1 occurs extracellularly, functioning as a damage-associated molecular pattern. This molecule binds to receptors such as TLR4 and RAGE, consequently activating downstream NFκB signaling and driving the transcription of pro-inflammatory cytokines, including IL-1β and IL-6. This cascade could amplify inflammatory injury in the myocardium by promoting immune cell infiltration and local cytokine release.

## Conclusion

The present findings lend support to the hypothesis that HMGB1 contributes to the early inflammatory response after MI, and that its inhibition can attenuate cytokine elevation without altering systemic morphological metrics. These findings contribute to a mounting body of evidence that the targeting of upstream HMGB1, could be a promising adjunctive strategy to mitigate MI-induced inflammation and enhance cardiac healing.

## Figures and Tables

**Table 1:** Nucleotide sequences of the primers used to quantify the expression of the genes Toll-like receptor (TLR), high mobility group box 1 (HMGB1), inducible NOS (iNOS), endothelial NOS (eNOS), superoxide dismutase (SOD), nuclear factor-κB p65 (RelA), interleukin 1

Gene		Primers	Amplicon size (bp)	T_m_ (°C)
***TLR4***	forwards	CAT GGC ATT GTT CCT TTC CTG C	119	60
NM_019178.2	reverse	GGA TGT CAT GAG GGA TTT TGC TG		
***HMGB1***	forwards	AGT GAG GGA GAG AGT GGG TAA A	137	59
NM_012963.4	reverse	CCT TCT GAT CTT GGC TCC TCA T		
***iNOS***	forwards	AAA CGC TAC ACT TCC AAC GC	91	59
NM_012611.3	reverse	TGC TGA GAG CTT TGT TGA GGT C		
***eNOS***	forwards	GAT CCC CCG GAG AAT GGA GA	105	60
NM_021838.2	reverse	TCG GAT TTT GTA ACT CTT GTG CT		
***SOD 1***	forwards	CTG AAG GCG AGC ATG GGT TC	131	60
NM_017050.1	reverse	TCC AAC ATG CCT CTC TTC ATC C		
***SOD 2***	forwards	GCT GGC CAA GGG AGA TGT TAC	83	60
NM_017051.2	reverse	TGC TGT GAT TGA TAT GGC CCC		
***RelA (NFκB)***	forwards	TCT GCC GAG TAA ACC GGA AC	93	61
NM_199267.2	reverse	ACA CCT CAA TGT CTT CTT TCT GC		
***IL-1β***	forwards	CAC CTC TCA AGC AGA GCA CAG	79	60
NM_031512.2	reverse	GGG TTC CAT GGT GAA GTC AAC		
***TNFα***	forwards	CGT CAG CCG ATT TGC CAT TTC	116	60
NM_012675.3	reverse	TGG GCT CAT ACC AGG GCT T		
***GPX4***	forwards	TAA GTA CAG GGG TTG CGT GTG	135	60
NM_017165.4	reverse	CAA GGG AAG GCC AGG ATT CG		
***Rpl10a***	forwards	TCC ACC TGG CTG TCA ACT TC	134	60
NM_031065.1	reverse	GGC AGC AAC GAG GTT TAT TGG		

β (IL-1β), tumor necrosis factor α (TNFα), glutathione peroxidase 4 (GPX4) and 60 S ribosomal protein L10a (Rpl10a).

Abbreviations: Tm, melting temperature; bp, DNA base pairs
